# Gypenosides Altered Hepatic Bile Acids Homeostasis in Mice Treated with High Fat Diet

**DOI:** 10.1155/2018/8098059

**Published:** 2018-07-12

**Authors:** Yanliu Lu, Yimei Du, Lin Qin, Di Wu, Wei Wang, Lei Ling, Feifei Ma, Hua Ling, Li Yang, Changhong Wang, Zhengtao Wang, Xumei Zhou, Yuqi He

**Affiliations:** ^1^Key Lab of the Basic Pharmacology of the Ministry of Education, Zunyi Medical University, 6 West Xue-Fu Road, Zunyi City, Guizhou 563009, China; ^2^Shanghai Key Laboratory of Complex Prescription, Shanghai University of Traditional Chinese Medicine, 1200 Cai-Lun Road, Shanghai 201203, China; ^3^School of Pharmacy, Zunyi Medical University, 6 West Xue-Fu Road, Zunyi City, Guizhou 563009, China; ^4^School of Pharmacy, Georgia Campus-Philadelphia College of Osteopathic Medicine, 625 Old Peachtree Rd NW Suwanee, GA 30024, USA

## Abstract

Gypenosides extracted from* Gynostemma pentaphyllum *(Thunb.) Makino have significant role in reducing serum lipid level and treating fatty liver diseases, however, without clear mechanism. As gypenosides share the similar core structures with bile acids (the endogenous ligands of nuclear receptor FXR), we hypothesize that gypenosides may improve hypercholesterolemia via FXR-mediated bile acids signaling. The present study was designed to validate the role of gypenosides in reducing levels of serum total cholesterol (TC) and low density lipoprotein cholesterol (LDL-C), as well as in regulating bile acids homeostasis and related gene expression levels. The C57BL/6 male mice were divided into four groups. Mice in groups ND and HFD were fed with normal diet and high fat diet for 38 weeks, respectively. In groups HFD+GP and HFD+ST, mice were fed with high fat diet for 38 weeks and treated with gypenosides and simvastatin (positive control) from weeks 16 to 38, respectively. Serum TC and LDL-C levels were assayed by commercially available kits. Expression levels of genes were tested by the quantitative real-time PCR. The LC-MS/MS was applied to quantify major bile acids in mice livers. Our results showed that gypenosides significantly decreased serum TC and LDL-C levels. The gene expression level of* Shp* was downregulated while the levels of* Cyp7a1*,* Cyp8b1*,* Fxr*,* Lrh1*,* Jnk1/2*, and* Erk1/2* were upregulated by gypenosides. Indicated by LC-MS/MS technology, gypenosides increased the hepatic levels of several free bile acids and most taurine-conjugated bile acids while decreasing glycine-conjugated bile acids levels. In addition, gypenosides decreased the CA/CDCA ratio. Gypenosides may improve the abnormal lipid profile of HFD-fed mice via two pathways: (1) enhancing the bile acids biosynthesis from cholesterol; (2) decreasing the CA/CDCA ratio which is positively related to cholesterol absorption.

## 1. Introduction

Hypercholesterolemia is associated with various diseases in hepatic, enteric, and cardiovascular systems [1]. Medicines such as statins, bile acid sequestrates, and PSCK9 inhibitors have been used to treat hypercholesterolemia for years, however, accompanied by various adverse drug events [2].

Gypenosides are extracted from the plant* Gynostemma (G.) pentaphyllum (Thunb.) *Makino that is distributed in China, Korea, Japan, and other Southeast Asian countries [3, 4]. To data, gypenosides reduce serum cholesterol, triglycerides, and blood glucose levels without significant adverse drug effect [5–11]. At the time of writing, around 100 gypenosides have been identified from this plant, constructing a potential drug candidate's pool for drug discovery and development. However, as the mechanism of gypenosides action in treating these diseases is still unclear, there is little progress on drug screening and development from gypenosides so far.

Gypenosides share the core structure of dammarane-type triterpene which is similar to the core structures of endogenous bile acids. Bile acids mediate the absorption of fat and cholesterol and regulate the lipid and carbohydrate metabolisms [12, 13]. In the livers, cholesterol is biotransformed to various bile acids by a serials enzymes involving CYP7A1, CTP8B1, and CYP27A1 [14, 15]. This process is sensitively regulated by farnesoid X receptor (FXR), a nuclear receptor regulating lipid and carbohydrate homeostasis [16–18]. Thus, regulation of FXR-mediated bile acids homeostasis could be a potential mechanism explaining the effect of gypenosides on improving lipid and carbohydrate profiles. Gypenosides are recognized as the relatives of ginsenosides with similar core structures. A couple ginsenosides showed significant role in regulation of CYP7A1, a rate-limit enzyme of bile acids biosynthesis [19]. Guggulsterone, a compound isolated from an Indian plant and also with the similar structure of gypenosides core, was reported to be the ligand of FXR [20].

In the present study, we aimed to validate the role of gypenosides on cholesterol regulation and investigate their effect on hepatic bile acids homeostasis at both gene expression and bile acids quantification level. Once a new mechanism of action and enzyme target is identified in our study, screening of FXR ligands may become an effective way to develop new drugs from gypenosides. Since classic enterohepatic regulation of bile acids exists, some enteric genes regulating bile acids pool profile were also checked. An ultraperformance liquid chromatography coupled with a triple quadruple tandem mass spectrometry was used to quantify 14 major bile acids molecules.

## 2. Materials And Methods

### 2.1. Materials

Gypenosides (purity > 98% assayed by UV) were purchased from Zhongxin Biotechnology Ltd. (Xian, Shanxi Province, China). Simvastatin tablets (20 mg/Tablet) were purchased from Xinqi Pharmaceutical Ltd. (Caoxian, Shandong Province, China). Normal diet (23.2% of calories from protein, 12.1% from fat, and 64.7% from carbohydrate) and high fat diet (20.0% of calories from protein, 60.0% from fat, and 20.0% from carbohydrate) were purchased from Research Diets Inc. (New Brunswick, NJ). DEPC water was purchased from Zoman Biotechnology Co., Ltd. (Beijing, China).

### 2.2. Quality Check of Gypenosides

An HPLC-PDA system was used to check the quality of gypenosides fed to mice in the present study. Gypenosides (250 mg) were dissolved in methanol (10 mL) to prepare the sample solution. An aliquot of 10 *μ*L of the sample solution was injected into a Synergi 4 *μ* MAX-RP 80R column (250 × 4.6 mm, 4 *μ*m) equipped in HPLC system (Agilent 1260, Santa Clara, CA) for separation. The column temperature and the flow rate were set at 30°C and 0.6 mL/min, respectively. Water (A) and acetonitrile (B) were used for elution with the following gradient: 0-1 min, A:B = 70:30; 1-2 min, A:B = 65:35; 2-22 min, A:B = 50:50; 22-38 min, A:B = 20:80; 38-45 min, A:B = 10:90; 45-46 min, A:B = 5:95. A PDA detector was used with wavelength ranging from 190 nm to 400 nm. Both the chromatograms and the UV spectrums were recorded.

### 2.3. Animal Experiments

Male C57BL/6 mice (23-25 g) were purchased from Huafu Kang Biological Technology Ltd. (Beijing, China; approval number: SCXK 2014-0004). All the animals were kept in the well-controlled animal room on the SPF level. Room temperature was set to 21 - 23°C, while the humidity was set to 50%-60%. A 12-hour light/dark cycle was used and animals were free to access food and water. Mice were divided into 4 groups (*n* = 5-10 per group). Two of these groups were treated with normal diet (ND) and high fat diet (HFD) for 38 weeks, respectively. The other two groups were treated with HFD for 38 weeks while orally being treated with GPs (HFD+GP, 250 mg/Kg) and simvastatin (HFD+ST, 20 mg/Kg) from weeks 16 to 38, respectively. The group treated with simvastatin was used as the positive control for lipid regulation. In our previous pilot study, the hypercholesterolemia and hyperglycemia model could be established at week 16 of HFD treatment. To confirm this time point, we used additional two groups of mice. These two additional groups of mice were treated with normal diet and high fat diet for 16 weeks, respectively. Mice in HFD+GP and HFD+ST groups were given gypenosides and simvastatin daily, respectively, while mice in ND and HFD groups were given 0.1% of CMC-Na solution (medium used to suspend gypenosides and simvastatin in water) with the equivalent volume. At the end point of the experiment, mice were anesthetized by 7% chloral hydrate (0.05 mL/10g). Whole blood, livers, and small intestines were collected or harvested. Blood was centrifuged at 3,500 rpm for 10 min after an incubation at room temperature for 1 hour to isolate serum. Once harvested, livers were cut to small pieces. One piece of liver was fixed in 10% formalin for pathological examination. All of the other samples were transferred to liquid nitrogen immediately for a quick frozen and transferred to -80°C freezer for long term storage. All procedures involving the use of laboratory animals were in accordance with the requirements of Animal Experiment Ethics Committee of Zunyi Medical University.

### 2.4. Histopathology Examination of Liver Tissues

After 24 hours, the fixed liver tissues were embedded in the wax (Leica EG1150, Wetzlar, Germany) and then cut into slices of 6–8 *μ*m by a Leica RM2245 Biosystems (Wetzlar, Germany). Tissue slices were washed, dehydrated, and stained by hematoxylin and eosin (HE) for microscopic examination (Olympus BX43, Tokyo, Japan, × 40).

### 2.5. Evaluation of Lipid Profiles in Serum

Serum total cholesterol (TC) and low density lipoprotein cholesterol (LDL-C) were determined by using kits purchased from Nanjing Jiancheng Bioengineering Institute (Nanjing, Jiangsu Province, China).

### 2.6. Expression Levels of Genes Involved in Bile Acids Regulation

Total RNA from mouse liver tissues and duodenum were extracted following our previously used procedures. Liver tissues of around 20 mg were thawed in 1mL of Trizol reagent. After homogenizing on ice, the homogenates were centrifuged and the upper layer was washed by 200 *μ*L of chloroform. Then 500 mL of isopropanol was used to precipitate RNA. Precipitates were washed with 75% of alcohol and then dissolved with 30 *μ*L of DEPC water after evaporating all alcohol. A Nanodrop spectrophotometer was used to quantify RNA samples. For each sample, 1 *μ*g of RNA was used to make cDNA following the procedure indicated by PrimeScript RT reagent kit (Company, City, TaKaRa, Japan) on a Mastercycler Gradient PCR Thermal cycler (Eppendorf Scientific, Inc., Germany). The temperature program for reverse transcription was set as follows: 25°C for 10 min, 37°C for 2 hours, and 85°C for 5 min.

The real-time polymerase chain reaction (RT-PCR) was performed by using 2^*∗*^SYBR Green Supermix (Bio-Rad, Germany) on a CFX96 RT-PCR System (C1000 Touch, BioRad, Germany). The RT-PCR parameters were as follows: 3 min, 95°C, and 1 cycle; 10 s at 95°C plus 45 s at 60°C, 39 cycles. The melting curve analysis consisted of 5 s at 55°C followed by heating up to 95°C with a ramp rate of 0.5°C / 5 s. Names and corresponding primer sequences of genes checked in the present study were shown in Table 1. Expression levels of GAPDH in each sample were used as internal reference.

### 2.7. Quantification of Hepatic Bile Acids

Approximately 100 mg of liver tissues was homogenized in 300 *μ*L of acetonitrile on ice. The homogenates were centrifuged at 14,300 rpm and 4°C for 10 min. An aliquot of 250 *μ*L of supernatants was evaporated by nitrogen gas blow and dissolved in 50% of methanol (100 *μ*L). After centrifugation at 14,300 rpm and 4°C for 10 min, the supernatants were used for quantification.

Quantifications of BAs were performed on a Triple Quad UPLC-MS/MS system (Agilent 6420, Santa Clara, CA). A CQUITY UPLC BEH C18 (1.7 *μ*m, 100 × 2.1 mm) column was used for BAs separation. The chromatographic parameters were modified from the published method [15]. The column compartment was maintained at 45°C. The mobile phases consisted of solvents A (10 mM of ammonium acetate in 20% acetonitrile) and B (10 mM of ammonium acetate in 80% acetonitrile). The elution gradient program was set as follows: 5% of B from 0 to 5 min; 5-14% of B from 5 to 14 min; 14–25% of B from 14 to 14.5 min; 25% of B from 14.5 to 17.5 min; 25-50% of B from 17.5 to 18 min; 50% of B from 18 to 22 min; 50-80% of B from 22 to 22.5 min; 80% of B from 22.5 to 24.5 min; 80-100% of B from 24.5 to 25 min; 100% of B from 25 to 27 min; 100-5% of B from 27 to 28 min; and 5% of B from 28 to 33 min. The injection volume was 10 *μ*L, and the flow rate was set at 0.3 mL/min. Parameters of the ESI ion source were as follows: negative mode; gas temp: 326°C, gas flow: 12 L/min, nebulizer pressure: 55 psi, and capillary voltage: 3500 V. The mass analyzer was set as the SIM mode to capture the [M-H]^−^ ions of expected BAs. Peak areas of each observed BAs were used for comparison and statistical analysis among groups.

### 2.8. Statistical Analysis

All data were shown as mean ± SEM. Statistical analyses between two groups were performed using Student's* t*-test while multiple comparisons were performed using one-way ANOVA in the SPSS Statistics 18.0 (IBM, Chicago, USA). A* p* value less than 0.05 was considered as statistically significant. Principal component analysis (PCA) was performed using the “pca” function in mixOmics package of the R program (i386, 3.3.3).

## 3. Results

### 3.1. Quality Analysis of The Gypenosides Used in the Present Study

A fingerprint of gypenosides was obtained from a HPLC-DAD system (Supplemental File 1). In total, 35 peaks were observed with S/N ratio values more than 10. Most peaks showed UV absorption patterns of end absorption, which were consistent with the properties of gypenosides. Represented UV spectrums of peak 7, peak 10, peak 20, peak 21, peak 24, peak 25, peak 26, and peak 30 were showed in part B of Supplemental File 1. All of these peaks had the relatively high amount in the gypenosides indicated by the peak area values. The purity of the gypenosides was more than 98% assayed by the ultraviolet spectrometry.

### 3.2. Preparation of Hypercholesterolemia Model

**Figure 1:**
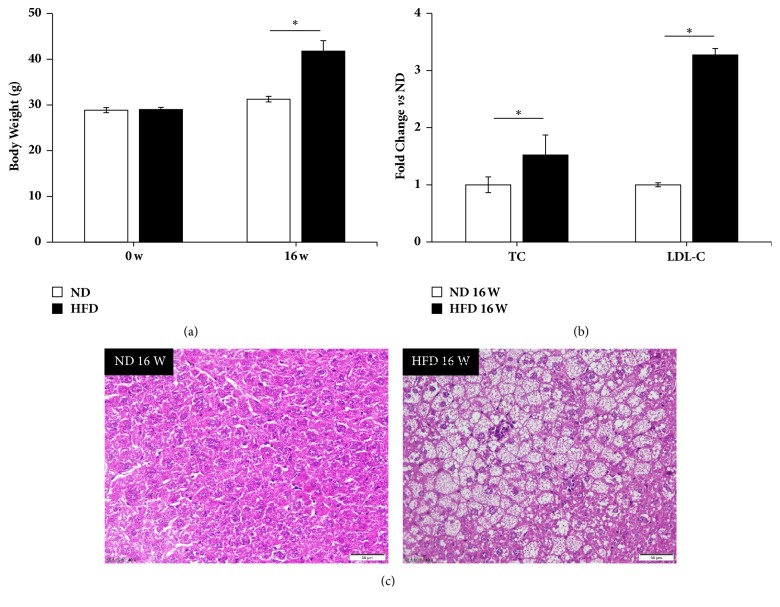
High fat diet induced hypercholesterolemia mouse models and fatty livers. (a) Mice body weight levels were increased significantly by high fat diet at week 16. (b) Serum total cholesterol (TC) and low density lipoprotein cholesterol (LDL-C) levels were induced by high fat diet at week 16. (c) HE staining of liver tissues showed an obvious lipid accumulation in hepatocytes of mice treated by high fat diet for 16 weeks. The images were shown as 40 times enlargement. W: week; ND: normal diet group; HFD: high fat diet group. Data were presented as mean ± SEM. ^*∗*^*p* < 0.05.

The high fat diet treatment significantly increased mouse body weight levels after 16 weeks (Figure 1(a)). In addition, TC and LDL-C levels were also increased by high fat diet (Figure 1(b)). In mouse livers, fat accumulation was observed (Figure 1(c)) and the portal area and central vein showed diffuse adipose hollow spaces (Figure 1(c)). Therefore, the hypercholesterolemia model was confirmed for this study.

### 3.3. Effects of Gypenosides on Lipid Regulation

**Figure 2:**
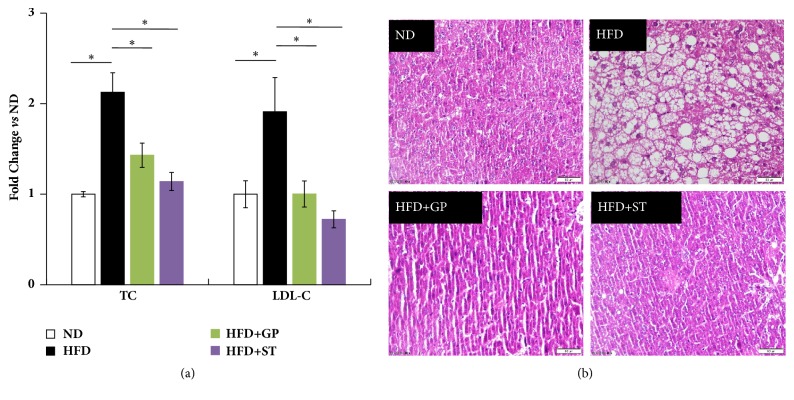
Gypenosides reversed the abnormal mouse serum and hepatic lipid homeostasis. (a) Both gypenosides and simvastatin reduced the serum total cholesterol (TC) and low density lipoprotein cholesterol (LDL-C) levels in serum of mice treated with high fat diet. (b) Both gypenosides and simvastatin reversed the trend of lipid accumulation in mice livers. HE staining images were enlarged by 40 times. ND: normal diet group; HFD: high fat diet group; HFD+GP: gypenosides treatment on mice fed with high fat diet; HFD+ST: simvastatin treatment on mice fed with high fat diet. Data were presented as mean ± SEM. ^*∗*^*p* < 0.05.

High fat diet significantly increased the TC and LDL-C levels in mouse serum (Figure 2(a)). After treatment, gypenosides and simvastatin successfully decreased the abnormally elevated TC and LDL-C levels. In addition, HE staining showed that the lipid accumulation in mouse hepatocytes was observed before treatment and then disappeared in mouse livers after receiving gypenosides (Figure 2(b)). These findings suggested that gypenosides may play a role in the lipid regulation, in particular on cholesterol reducing.

### 3.4. Gypenosides Altered the Hepatic Gene Expression Levels pf* Cyp7a1* and* Cyp8b1*, Genes Encoding Enzymes Limiting Rate of Bile Acids Biosynthesis

**Figure 3:**
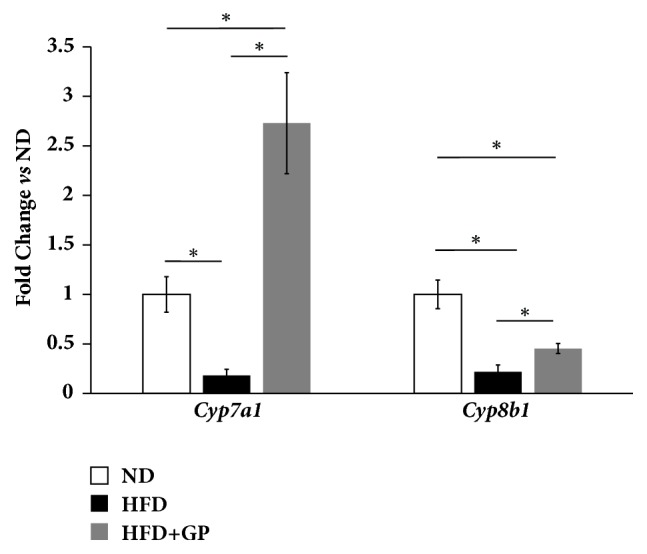
Gypenosides changed the gene expression levels of mouse hepatic* Cyp7a1* and* Cyp8b1* which were two rate-limit enzymes of bile acids biosynthesis. ND: normal diet group; HFD: high fat diet group; HFD+GP: gypenosides treatment on mice fed with high fat diet. Data are presented as mean ± SEM. ^*∗*^*p* < 0.05.

High fat diet reduced the hepatic gene expression levels of* Cyp7a1 and Cyp8b1 *by times. The livers of mouse treated with gypenosides showed a significantly higher gene expression of* Cyp7a1* compare to HFD group and the ND group (Figure 3). Gypenosides also increased the hepatic gene expression level of* Cyp8b1 *in mouse treated with high fat diet; however, they did not recover it to the level of ND group.

### 3.5. Gypenosides Altered the Hepatic Expression Levels of Genes Involved in Hepatic Regulation Pathway of Bile Acids Homeostasis

**Figure 4:**
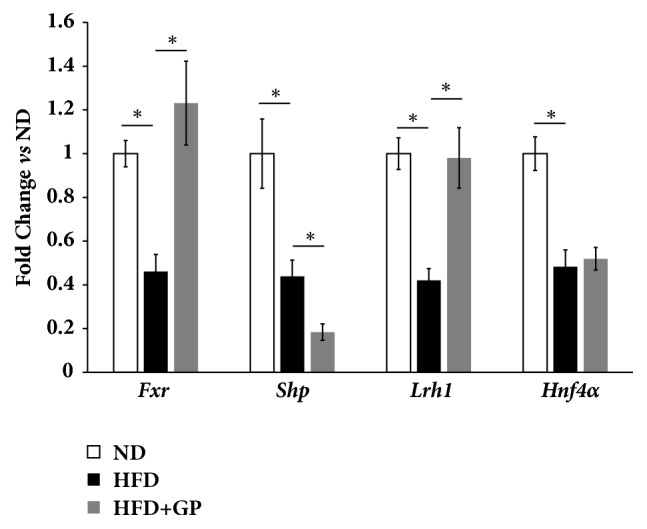
Gypenosides changed the expression levels of genes involved in hepatic pathway of bile acids homeostasis regulation. ND: normal diet treatment; HFD: high fat diet group; HFD+GP: gypenosides treatment on mice fed with high fat diet. Data are presented as mean ± SEM. ^*∗*^*p* < 0.05.

*Fxr, Shp, Lrh1, *and* Hnf4α* encode four important factors regulating bile acids homeostasis in mouse livers. High fat diet treatment widely inhibited the expression levels of these mouse hepatic genes. Gypenosides recovered the gene expression levels of* Fxr* and* Lrh1* to the levels of mice in ND group (Figure 4). However, gypenosides failed to change the gene expression levels of mouse hepatic* Hnf4α* in mice treated with high fat diet. Notably, gypenosides repressed the gene expression levels of* Shp, *an important participator of bile acids biosynthesis regulation, compared to HFD group.

### 3.6. Gypenosides Altered the Expression Levels of Genes Involved in Enterohepatic Regulation Pathway of Bile Acids Homeostasis

**Figure 5:**
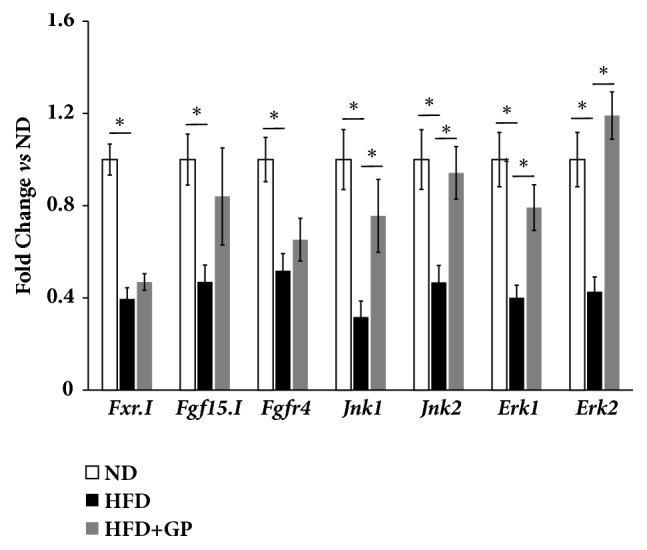
Gypenosides changed the expression levels of genes involved in enterohepatic pathway of bile acids homeostasis regulations. ND: normal diet group; HFD: high fat diet group; HFD+GP: gypenosides treatment on mice fed with high fat diet. Data are presented as mean ± SEM. ^*∗*^*p* < 0.05.

In mice enterohepatic pathway, the mRNA levels of all investigated genes were significantly repressed by high fat diet (Figure 5). After treatment with gypenosides, gene expression of* Jnk1, Jnk2*,* Erk1*, and* Erk2* was recovered. However, gene expressions of intestinal* Fxr, Fgf15*, and hepatic* Fgfr4* were still lower than that in mice treated with normal diet.

### 3.7. Effects of Gypenosides on Relative Changes of Bile Acids in Mice Liver

**Figure 6:**
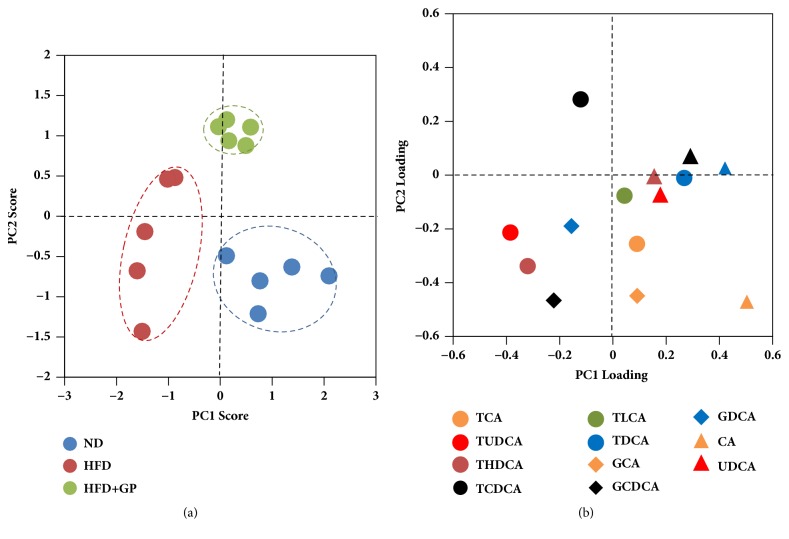
Gypenosides changed the global profile of mouse hepatic bile acids pool. (a) Score plots of principle component analysis on bile acids profiles. Each spot represents a mouse sample. Gypenosides made the location of mice shift away high fat diet group. ND: normal diet group; HFD: high fat diet group; HFD+GP: gypenosides treatment on mice fed with high fat diet; (b) loading plots of principle component analysis. Each spot represents a bile acid. Location of bile acids on loading plots represents its contribution on components.

**Figure 7:**
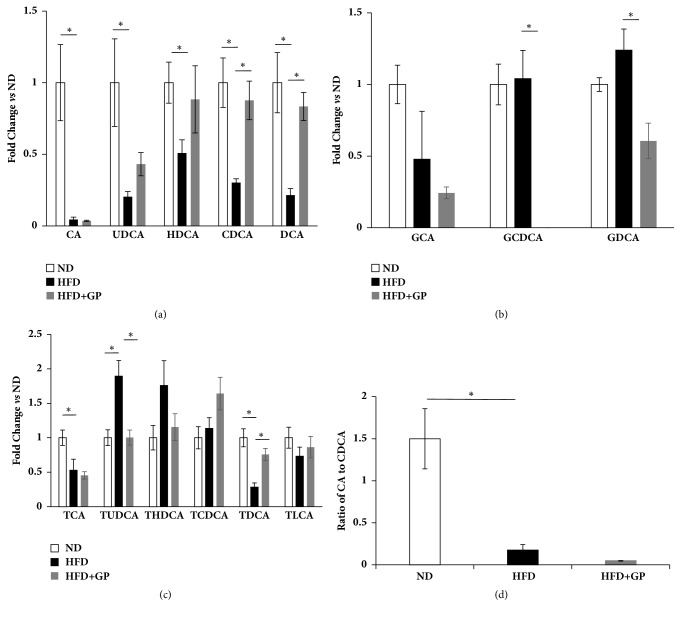
Mouse hepatic bile acids levels shown as histograms. (a) Gypenosides effect on free bile acids levels. (b) Gypenosides effect on glycine-conjugated bile acids levels. (c) Gypenosides effect on taurine-conjugated bile acids levels. (d) Gypenosides effect on CA/CDCA ratio. ND: normal diet group; HFD: high fat diet group; HFD+GP: gypenosides treatment on mice fed with high fat diet. Data were presented as mean ± SEM. ^*∗*^*p* < 0.05.

In the present study, 14 bile acids were quantified in mice livers using LC-MS/MS system. A principle component analysis on bile acids data showed that high fat diet significantly altered the hepatic bile acids profiles (Figure 6(a)). Component 1 contributed the most to the location of each mouse. Mice treated with high fat diet shifted along component 1. After treatment with gypenosides, mice showed a return trend along component 1; however, they showed a significant shift along component 2. According to loading plot (Figure 6(b)), TUDCA, THDCA, and GCDCA contributed more to HFD group, CA contributed more to ND group, and TCDCA contributed more to HFD+GP group. A regular histogram showed the similar trend of these aforementioned bile acids (Figures 7(a), 7(b), and 7(c)). In addition, CA/CDCA ratio is an important ratio to evaluate the liver function. Both high fat diet and gypenosides significantly inhibited the ratio. (Figure 7(d)).

## 4. Discussion

Animals fed with diet enriching high fat have been widely used as an experimental model to induce the hyperlipidemia syndrome [21–23]. In this study, total cholesterol, LDL-C, and mice body weight levels were significantly increased at week 16, demonstrating the reliability and reproducibility of this model. Compared to chemical-induced and transgenic animal model, high fat diet induced hyperlipidemia model modulates the related clinic syndromes more precisely [24–26].

Gypenoside is derived from* Gynostemma pentaphyllum *(Thunb.) Makino, a Chinese herbal medicine [27]. To data, gypenosides ARE reported to reduce the hepatic TC levels of the hyperlipidemia rats; however, the mechanism is still not fully understood [28]. In this study, daily administration of gypenosides for 22 weeks significantly reduced the serum TC and LDL-C levels, comparable to the effect of simvastatin, a widely used medication for hypercholesterolemia [29]. Furthermore, gypenosides shared the similar core structures of bile acids, which are the endogenous ligands of nuclear receptor FXR, an important regulator of lipid and carbohydrates metabolism [30]. Our studies showed that gypenosides may reduce serum cholesterol and LDL-C levels via FXR signaling. Bile acids homeostasis is directly regulated by FXR via hepatic and enterohepatic pathways. In hepatic pathway, FXR induces SHP which inhibits LRH1 and HNF4*α* [31]. As LRH1 and HNF4*α* are direct inducers of CYP7A1 [32], the rate-limit enzyme of bile acids biosynthesis, stimulating FXR would eventually inhibit the generation of bile acids from cholesterol. On the other hand, once bile acids are excreted to small intestine via bile duct, intestinal FXR would be stimulated to enhance the secretion of Fgf15 [33]. The latter is transported back to liver via the port vein to bind with hepatic FGFR4, which will subsequently induce* Jnk/Erk *to inhibit gene expression of* Cyp7a1* [34]. In the present study, in both hepatic and enterohepatic pathways, we checked the expression of key genes involving hepatic* Cyp7a1*,* Cyp8b1*,* Fxr*,* Shp*,* Lrh1*,* Hnf4α*,* Jnk1/2*,* Erk1/2*, and* Fgfr4*, as well as intestinal* Fxr* and* Fgf15*. Data confirmed that gypenosides significantly altered the expression levels for most of these genes. Thus, the study results supported our hypothesis that FXR may be one of the target of gypenosides in mice livers.

CYP7A1 is one of the rate-limiting enzymes involved in the classic pathway of bile acid biosynthesis. Hepatic SHP represses the transcription of CYP7A1 by competing with the nuclear receptor liver receptor homolog 1 (LRH1), which is a constitutive activator of the* Cyp7a1 *gene [35]. In this study we observed that gypenosides decreased the hepatic gene expression levels of* Shp* in hyperlipidemia mice while they increased the gene expressions of* Cyp7a1 *and* Lrh1*. This implied that gypenosides may enhance the biosynthesis of bile acids from cholesterol. Actually, in GP-treated mice, we observed the increased levels for several abundantly available hepatic bile acids such as CDCA, DCA, and TDCA. Thus, inhibition of* Shp* gene expression accompanied with induction of* Cyp7a1 *and* Lrh1 *gene expression and subsequently enhanced the cholesterol metabolism may be one of the mechanisms of gypenosides action in reducing serum cholesterol.

The microsomal CYP8B1 enzyme is essential for the biosynthesis of CA, which regulates CA/CDCA ratio [36]. In the present study, although gypenosides induced* Cyp8b1*, the level was still not fully restored. Thus, hepatic CA levels were still extremely low. On the other hand, the hepatic CDCA level was higher in the group treated with gypenosides compared to HFD group. Eventually, GP downregulated the CA/CDCA ratio compared to both ND and HFD groups. To data, CA/CDCA ratio is associated with enhanced cholesterol absorption and hypercholesterolemia [37]. Therefore, restricting the cholesterol absorption may be the other mechanism that explains the impact of gypenosides in reducing mice serum cholesterol levels.

Accumulation of bile acids leads to hepatic damage [38]. Enhanced transformation from cholesterol to bile acids would potentially lead to the accumulation of bile acids in hepatocytes causing hepatic damage. However, liver damage was not observed in gypenosides-treated mice. The possible explanation is that free bile acids were subsequently transformed to conjugated bile acids like glycine and taurine conjugation. Glycine-conjugated bile acids may induce reactive oxygen species and eventually lead to hepatocyte apoptosis [39]. In the present study, gypenosides kept the pathway of taurine conjugation on bile acids open but restricted the glycine conjugation on bile acids. This may be an advantage of medications developed from gypenosides pool.

## 5. Conclusion

Gypenosides significantly improved the abnormal lipid profile in mice treated with high fat diet, while genes regulating bile acids homeostasis were also changed. Gypenosides may improve the abnormal lipid profile of HFD-treated mice via two pathways: (1) enhancing the bile acids biosynthesis from cholesterol; (2) decreasing the CA/CDCA ratio which is positively related to cholesterol absorption. As bile acids regulation is the most sensitive signaling pathway of nuclear receptor FXR, FXR may be the potential therapeutic target of gypenosides.

## Figures and Tables

**Table 1:** Primers for real-time PCR assays.

Gene	Forward Primers	Reverse primers
*Cyp7a1*	GAGCCCTGAAGCAATGAAAG	GCTGTCCGGATATTCAAGGA
*Cyp8b1*	GGACAGCCTATCCTTGGTGA	GACGGAACTTCCTGAACAGC
*Nr1h4*	TTCCTCAAGTTCAGCCACAG	TCGCCTGAGTTCATAGATGC
*Nr0b2*	GGAGTCTTTCTGGAGCCTTG	ATCTGGGTTGAAGAGGATCG
*Nr5a2*	TCAGTTCGATCAGCGGGAGTTTGT	TGCAGGTTCTCCAGGTTCTTCACA
*Hnf4α*	GTGCTTCCGGGCTGGCATGAA	AGGTGATCTGCTGGGACAGAACC
*Mapk8*	CGTGGACTTATGGTCTGTGG	AGAGGATTTTGTGGCAAACC
*Mapk9*	TGCGATTGAAGAGTGGAAAG	TGAAGGCTGGTCTTTTACCC
*Mapk1*	TCAGTCCTTTTGAGCACCAG	TCATTTGCTCAATGGTTGG
*Mapk3*	TCCATCGACATCTGGTCTGT	AGCTGGTCCAGGTAGTGCTT
*Fgfr4*	GTCTCCGAGATGGAGGTGAT	CCAGCAGGTTGATGATGTTC
*Gapdh*	TGTGTCCGTCGTGGATCTGA	CCTGCTTCACCACCTTCTTGA

## Data Availability

All original data could be found in Supplemental File 2, or readers could directly send emails to HyqJeff@foxmail.com to contact Dr. Yuqi He to request the original data and accessing methods.

## Supplementary Materials

Supplementary 1 Quality analysis of gypenosides used in the present study. (A) High performance liquid chromatography (HPLC) chromatogram of gypenosides. (B) UV spectrum of represented peaks.

Supplementary 2 Original data of the present study.
