# Supplementary figures and images for: Gypenosides Altered Hepatic Bile Acids Homeostasis in Mice Treated with High Fat Diet
Source: Evid Based Complement Alternat Med. 2018 Jul 12;2018:8098059. doi: 10.1155/2018/8098059 (PMC6076974; doi:10.1155/2018/8098059)

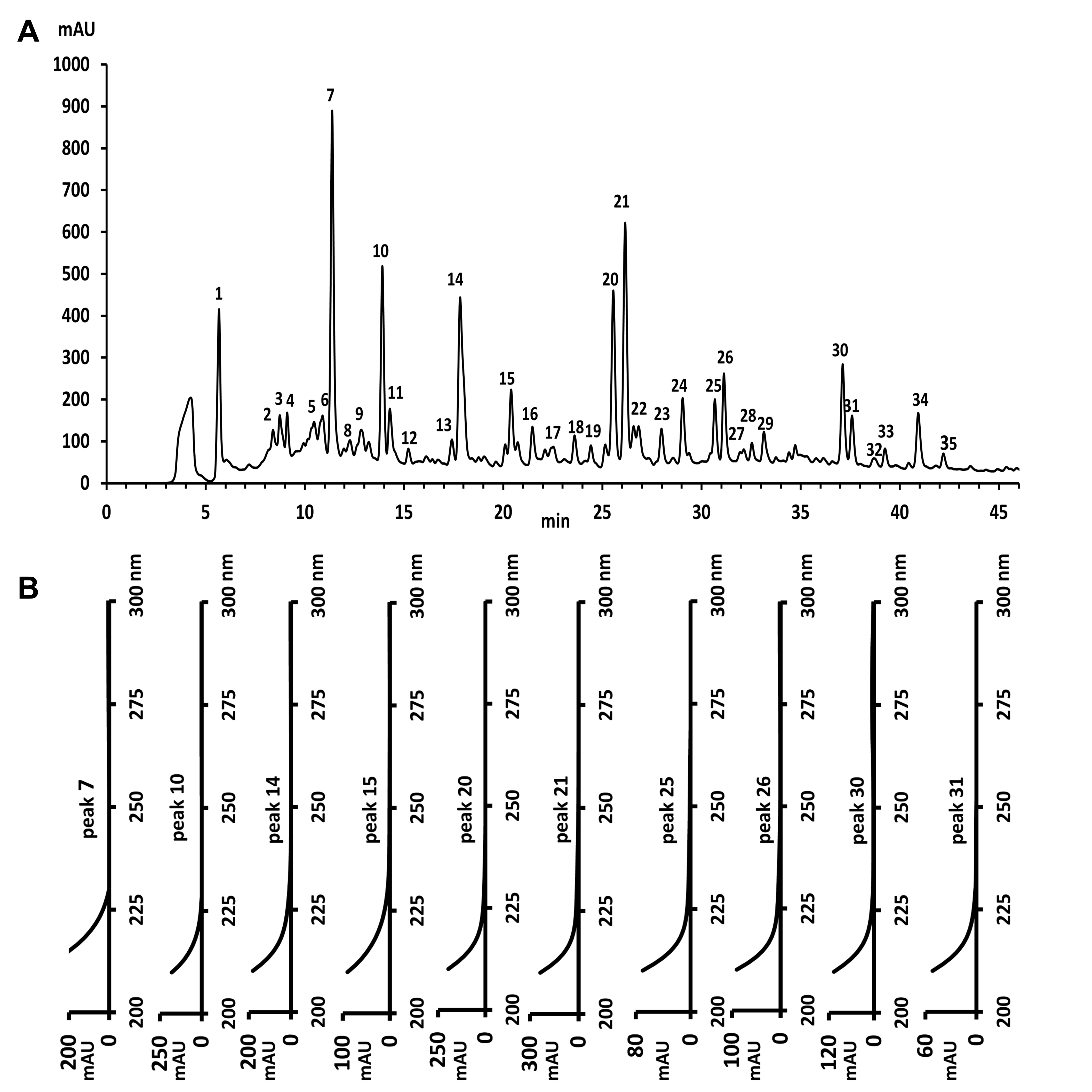

Supplement: Supplementary 1 — Quality analysis of gypenosides used in the present study. (A) High performance liquid chromatography (HPLC) chromatogram of gypenosides. (B) UV spectrum of represented peaks. [file 8098059.f1.tif]
